# Multimodal imaging in discriminating atrial septal defect combined with mobile echoic strands in fossa ovalis: a case report

**DOI:** 10.1093/ehjcr/ytag701

**Published:** 2026-09-17

**Authors:** Yi Cheng, Jing Li, Xiaojing Ma

**Affiliations:** Department of Ultrasound, Wuhan Asia General Hospital, School of Medicine, Wuhan University of Science and Technology, Wuhan 430056, China; Department of Ultrasound, Wuhan Asia General Hospital, School of Medicine, Wuhan University of Science and Technology, Wuhan 430056, China; Department of Ultrasound, Wuhan Asia General Hospital, School of Medicine, Wuhan University of Science and Technology, Wuhan 430056, China

**Keywords:** Multimodal imaging, Atrial septal defect, Fossa ovalis, Cystic haemangioma, Residual fragments of the primary septum, Thrombus, Case report

## Abstract

**Background:**

Mobile echoic structures in the fossa ovalis region represent a common diagnostic pitfall in patients with atrial septal defect (ASD). Misdiagnosis as pathological masses may lead to unnecessary invasive interventions. This case demonstrates the value of a stepwise multimodal imaging strategy for differential diagnosis.

**Case summary:**

A 48-year-old female presented with intermittent chest discomfort and was initially diagnosed with suspected patent foramen ovale (PFO) at a referring hospital. After systematic evaluation at our centre, the diagnosis was revised to secundum ASD (fossa ovalis type) accompanied by a mobile linear hyperechoic structure at the defect margin. The patient underwent transoesophageal echocardiography (TEE), transthoracic contrast echocardiography, and contrast-enhanced cardiac computed tomography, with complete laboratory workup to exclude infectious and thrombotic aetiologies.

**Discussion:**

High-resolution TEE confirmed a true anatomical septal defect rather than a PFO flap channel. Multimodal imaging stepwise excluded pathological mimics, including infective vegetation, mural thrombus, and cystic haemangioma, and the lesion was finally identified as benign residual fragments of the primary septum. Accurate differentiation directly guided clinical decision-making and avoided unnecessary invasive intervention.

Learning points

- Mobile linear echoes at the fossa ovalis margin in patients with secundum atrial septal defect (ASD) most commonly represent benign residual primary septum fragments and should be prioritized in differential diagnosis.
- A stepwise multimodal imaging strategy, including transoesophageal echocardiography, contrast echocardiography, and cardiac computed tomography enables reliable differentiation of benign septal remnants from pathological mimics.
- Confirmed primary septal remnants require no additional resection during ASD closure, as they are not associated with increased thromboembolic or endocarditis risk.

## Introduction

Secundum atrial septal defect (ASD) is a common congenital heart disease, and mobile linear echoic strands at the fossa ovalis margin are frequently encountered during pre-procedural echocardiographic evaluation. Without multimodal imaging, these structures are often misinterpreted as pathological masses, such as infective vegetation, mural thrombus, or cardiac haemangioma, which may result in unnecessary surgical resection, inappropriate anticoagulation, or delayed appropriate intervention.

## Summary figure

**Figure:**
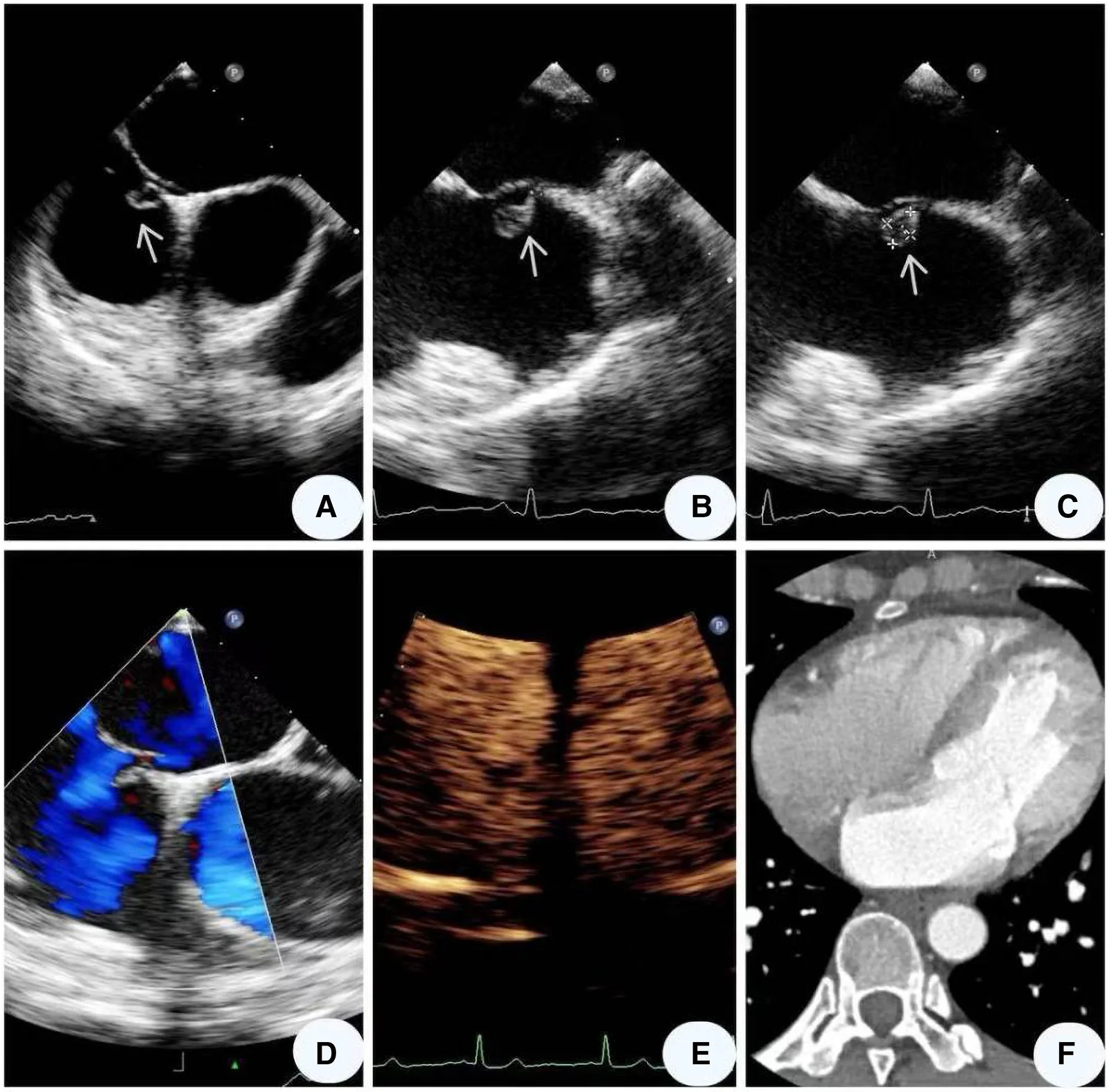


Although multimodal imaging is widely applied in structural heart disease assessment, few reports have systematically described a stepwise diagnostic workflow for fossa ovalis mobile lesions combined with embryological reasoning. This case presents a comprehensive multimodal evaluation process for a patient with secundum ASD and mobile septal strands and highlights its clinical impact on treatment decision-making.

## Case report

A 48-year-old female presented to our department for further evaluation of intermittent chest discomfort of 1 month duration. She had initially consulted a local hospital in Xiangyang City for her symptoms, where transthoracic echocardiography suggested a possible patent foramen ovale (PFO) with Grade III shunt. She was subsequently referred to our centre for definitive diagnosis and surgical planning.

She had no history of hypertension, diabetes mellitus, or food or drug allergies. Her past surgical history included uterine myomectomy and total hysterectomy. She had no history of fever, recent systemic infection, or intravenous drug use. She reported no regular long-term medication use. Her baseline blood pressure was chronically low without symptoms of dizziness or fatigue, which was considered constitutional hypotension.

Physical examination revealed clear breath sounds in both lungs without rales, a regular heart rate of 70 beats per minute, no pathological murmurs at any valve auscultation areas, a blood pressure of 88/63 mmHg, a soft abdomen without tenderness or rebound tenderness, no hepatosplenomegaly, and no lower extremity oedema.

Laboratory examinations showed normal white blood cell count and neutrophil percentage. C-reactive protein and procalcitonin levels were within normal limits, indicating no active systemic inflammation. Three sets of blood cultures collected at different time points all showed negative results after 5 days of incubation, ruling out active bactereamia. Coagulation function and D-dimer levels were also normal, with no evidence of a hypercoagulable state. Additional tests, including liver and renal function, myocardial enzymes, and N-terminal pro-B-type natriuretic peptide, were all within normal reference ranges.

Transoesophageal echocardiography (TEE) was performed for definitive anatomical assessment. Initial transthoracic echocardiography at the referring hospital had misdiagnosed the lesion as PFO due to limited acoustic window, with the mobile septal remnant mimicking a PFO flap valve. In contrast, high-resolution biplane TEE clearly demonstrated deficiency of the septum secundum muscular rim at the fossa ovalis, confirming a true anatomical tissue defect rather than a potential flap channel. The diagnosis was therefore revised to secundum ASD (fossa ovalis type), in accordance with the 2020 European Society of Cardiology (ESC) Guidelines for the management of adult congenital heart disease.^13^

Colour Doppler flow imaging revealed a Grade III massive left-to-right shunt. Shunt severity was graded using the standard echocardiographic grading system for intracardiac shunts (Grade I, trace; Grade II, mild; Grade III, massive), defined by the width of the shunt jet and the degree of right heart chamber opacification, consistent with the American Society of Echocardiography guidelines.^1^

The maximum defect diameter measured 0.9 cm on the biatrial view at end systole; this value varied slightly with respiratory phase and right atrial pressure and served as the core parameter for transcatheter closure device sizing. At the margin of the fossa ovalis defect, a linear hyperechoic structure was visualized (*Figure 1A–C*), measuring 0.53 cm × 0.75 cm with a 0.21-cm-wide pedicle. The structure oscillated regularly with the cardiac cycle, and color doppler flow imaging (CDFI) detected only minimal peripheral blood flow signal without internal vascularity.

**Figure 1:**
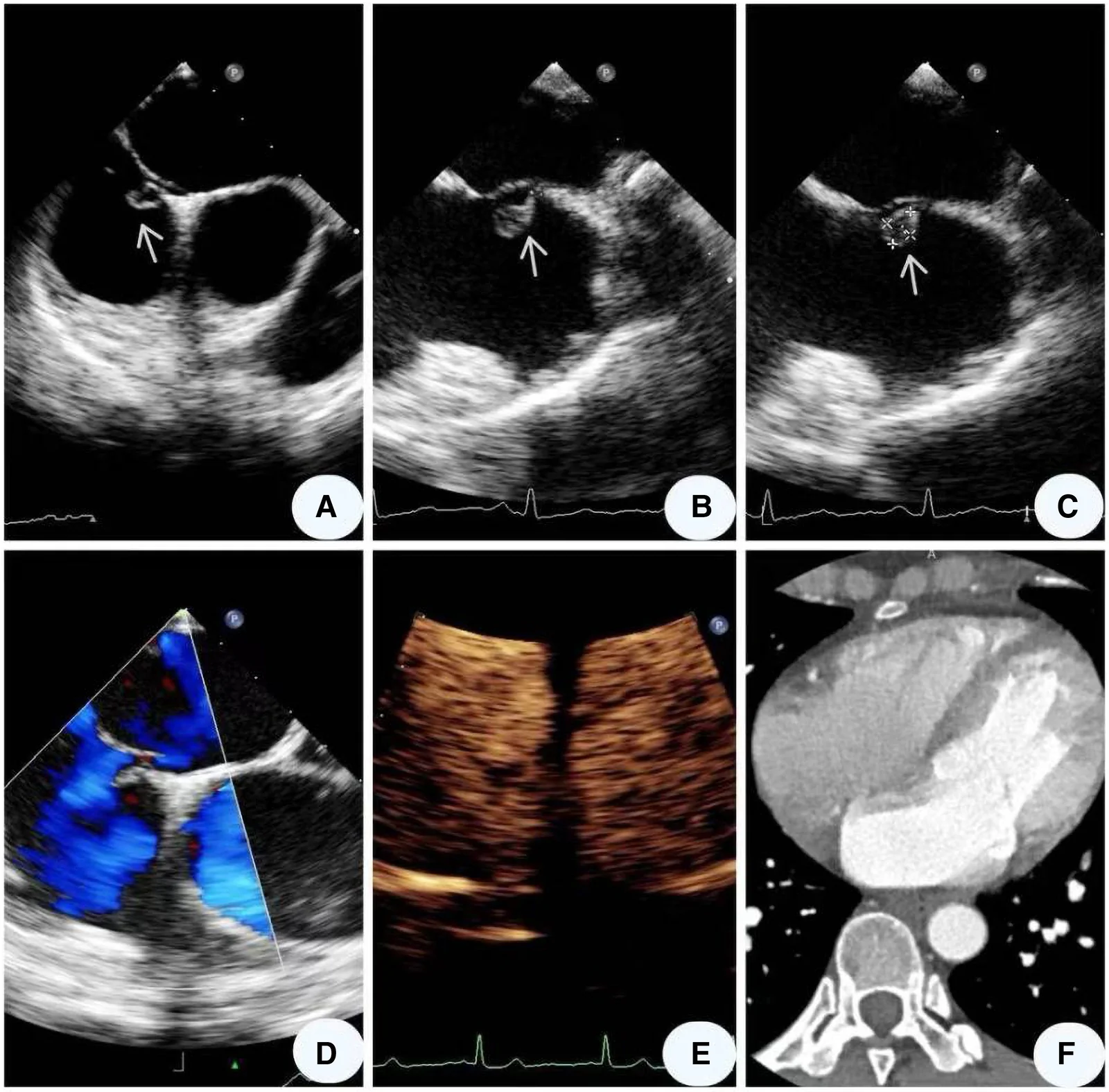
Six-panel composite figure showing multimodal imaging of an atrial septal defect. (*A–D*) Transoesophageal echocardiography views of the fossa ovalis defect with a mobile linear hyperechoic structure at the margin. (*E*) Contrast echocardiography with no internal enhancement within the lesion. (*F*) Contrast-enhanced cardiac computed tomography confirming the defect and homogeneous soft tissue density of the septal strand.

Transoesophageal echocardiography further showed normal right atrial and right ventricular dimensions (right ventricular basal diameter 3.2 cm), no interventricular septal flattening, and an estimated pulmonary artery systolic pressure of 28 mmHg (within normal range). No secondary right heart remodelling was detected despite the significant shunt, which was attributed to the moderate defect size and long-term haemodynamic adaptation.

Left heart contrast echocardiography (*Figure 1E*) was performed via peripheral intravenous injection of an ultrasound contrast agent. After contrast opacification of the left atrium, no obvious contrast filling was observed in the central part of the linear lesion, with only thin annular enhancement at the lesion edge, indicating no internal microvascular structure within the strand.

Contrast-enhanced cardiac computed tomography (*Figure 1F*) was subsequently performed to further characterize the lesion and assist pre-procedural planning. CT confirmed the presence of a central ASD consistent with TEE findings. The linear structure on the septal margin showed soft tissue density identical to normal septal tissue, no nodular or cystic component, and no abnormal enhancement pattern typical of vascular tumours. No filling defect suggestive of thrombus was identified, and no other intracardiac structural abnormalities or mediastinal lesions were detected.

After multidisciplinary discussion, including cardiology, cardiac surgery, and radiology, the final diagnosis was established as secundum ASD (fossa ovalis type) combined with residual fragments of the primary septum. Based on the confirmed benign nature of the septal lesion, the clinical team determined that no additional resection of the structure was necessary during ASD repair, to avoid unnecessary procedural risk. The patient underwent uncomplicated transcatheter ASD closure. She recovered smoothly and was discharged 3 days after the procedure. At 3-month follow-up, transthoracic echocardiography showed complete closure of the ASD with no residual shunt, and the septal remnant remained unchanged in size and mobility without thrombus formation. The patient’s chest discomfort symptoms were completely resolved.

## Discussion

Mobile echoic structures in the fossa ovalis region in patients with ASD present a common diagnostic pitfall in clinical echocardiography. The imaging features of benign septal remnants and pathological lesions, such as thrombus, vegetation, and haemangioma, show substantial overlap on single-modality imaging. Misdiagnosis may lead to unnecessary surgical resection, inappropriate anticoagulation, or delayed treatment of true pathology. Therefore, comprehensive analysis combining embryological knowledge, multimodal imaging characteristics, and clinical context is required to reach an accurate diagnosis. This case demonstrates the value of a stepwise multimodal approach in distinguishing benign septal remnants from pathological lesions and provides reference for clinical management.

### Embryological basis and pathophysiology of fossa ovalis atrial septal defect and primary septal remnants

Normal development of the atrial septum involves sequential growth, fusion, and resorption of the septum primum (primary septum) and septum secundum (secondary septum) during embryonic Weeks 5–6.^1,2^ The septum primum first grows caudally from the superior atrial wall, separating the primitive atrium into left and right chambers. Its central portion gradually resorbs to form the ostium secundum, maintaining fetal right-to-left shunting. Subsequently, the septum secundum develops to the right of the septum primum, growing in a crescent shape and eventually forming the thick muscular rim of the fossa ovalis. The remaining thin flap of the septum primum forms the valve of the foramen ovale. After birth, with the increase of left atrial pressure, the valve functionally apposes to the septum secundum, and complete anatomical fusion occurs within 1 year of age in ∼75% of individuals.^3^

The core pathogenetic distinction between fossa ovalis type secundum ASD and PFO lies in the structural integrity of the septum secundum.^1^ Patent foramen ovale represents a failure of anatomical fusion between the intact septum primum flap and the septum secundum, forming a potential flap-valve channel with a complete muscular rim. In contrast, secundum ASD is a true anatomical tissue defect caused by hypoplasia or excessive resorption of the septum secundum, resulting in a permanent defect with a deficient or absent muscular rim.

In patients with septum secundum hypoplasia, irregular residual fragments of the septum primum are commonly retained along the defect margin. These remnants represent incompletely resorbed portions of the original septum primum fibrous tissue, which remain attached to the defective septal rim and exhibit passive mobility driven by *trans*-septal pressure gradients and blood flow during the cardiac cycle.^4,14^ Unlike pathological masses, these remnants are composed of normal endocardial and fibrous tissue with no independent vascular supply, which is consistent with the imaging findings in our case. This embryological perspective provides the foundational rationale for considering primary septal remnant as a benign diagnosis of exclusion.

### Expanded differential diagnosis of mobile fossa ovalis structures

For mobile echoic strands in the fossa ovalis region, a broad differential diagnosis must be considered, with systematic exclusion based on clinical, laboratory, and imaging features. Accurate differentiation among these entities has direct clinical implications: benign primary septal remnants require no additional intervention beyond ASD treatment, while pathological lesions require targeted therapies, such as anticoagulation, antibiotics, or surgical resection. Correct preoperative diagnosis prevents unnecessary invasive procedures and ensures appropriate individualized management.

*Table 1* summarizes the key differentiating features of the main differential diagnoses across multimodal imaging and clinical characteristics.

**Table 1:** Differential diagnostic features of mobile fossa ovalis lesions on multimodal imaging

Diagnosis	Morphology and echogenicity	Attachment pattern	Mobility	Vascularity (CDFI/contrast echo)	Contrast CT features	Associated clinical features
Residual primary septum	Smooth linear shape, uniform hyperechogenicity	Narrow pedicle attached to defect margin	Regular, passive oscillation with cardiac cycle	No internal blood flow; only marginal annular enhancement on contrast	Soft tissue density same as normal septum, no abnormal enhancement	None; benign anatomical variant
Infective vegetation	Irregular, villous shape, heterogeneous echogenicity	Broad base, often at jet impact site	Chaotic, independent high-amplitude mobility	Variable internal vascularity in active phase	Irregular soft tissue mass, heterogeneous enhancement	Fever, elevated inflammatory markers, positive blood cultures
Mural thrombus	Heterogeneous echotexture, hypoechoic in acute phase	Broad base attached to endocardium	Poor mobility, stiff texture	No internal blood flow; filling defect on contrast	Hypodense filling defect, no enhancement	Right atrial enlargement, hypercoagulable state, history of embolism
Cystic haemangioma	Mixed echogenicity with cystic components	Broad sessile attachment	Limited, low mobility	Abundant internal blood flow; reticular centripetal enhancement	Nodular marginal enhancement in arterial phase, progressive centripetal filling	Rare; usually asymptomatic when small

#### Infective vegetations

Infective vegetations are included as a mandatory theoretical differential diagnosis for any mobile intracardiac mass, even when clinical suspicion is low, as missed diagnosis of infective endocarditis carries severe thromboembolic and mortality risks.^7^ Vegetations may rarely involve the atrial septum, particularly in the setting of septal defects with high-velocity jet flow causing endothelial damage. Typical features include irregular shape, heterogeneous echogenicity, and independent, chaotic mobility. Clinically, patients almost universally present with fever, positive blood cultures, and elevated inflammatory markers. In our case, the patient had no history of fever or immunosuppression, serial inflammatory markers were normal, and three sets of blood cultures were persistently negative. The lesion had a smooth linear morphology with uniform hyperechogenicity and regular rhythmic oscillation, which was not consistent with the typical appearance of infective vegetations; thus, this diagnosis was reliably excluded.

#### Intracardiac thrombus

While thrombus formation is more frequently documented after ASD closure or repair, *de novo* mural thrombus can also develop at the margins of an unrepaired ASD. This occurs most commonly in the setting of right atrial enlargement, low-velocity blood flow stasis, or systemic hypercoagulability, with an estimated prevalence of 2%–5% in symptomatic adult ASD patients.^8^ Organized thrombi typically appear as stiff, poorly mobile masses with heterogeneous echotexture, and acute thrombi are often hypoechoic. In this case, the patient had no right atrial enlargement on imaging, normal coagulation function and D-dimer levels, and no clinical risk factors for thromboembolism. The free, rhythmic mobility of the lesion was inconsistent with organized thrombus, and contrast echocardiography showed no typical filling defect pattern, supporting exclusion of this diagnosis.

#### Cardiac haemangioma

Cardiac haemangiomas are rare benign primary cardiac tumours, accounting for ∼5%–10% of all benign cardiac neoplasms. Location in the interatrial septum is extremely uncommon, representing <5% of all cardiac haemangioma cases.^5,6^ Cystic haemangiomas typically present as hypoechoic or mixed echogenic masses with cystic components, with relatively limited mobility. CDFI shows abundant internal blood flow signal, contrast echocardiography demonstrates reticular or centripetal enhancement within the lesion, and contrast-enhanced computed tomography (CT) shows characteristic progressive centripetal filling on delayed phases.^5^ In our case, the lesion was uniformly hyperechoic without cystic components, with no internal blood flow on CDFI and no internal enhancement on contrast echocardiography. Contrast CT also showed no typical enhancement pattern of haemangioma, making this diagnosis highly unlikely.

#### Additional differential considerations

Further differential diagnoses include Chiari network, Lambl excrescences, and atrial septal aneurysm. Chiari network is a reticular embryonic remnant in the right atrium derived from the resorbed right sinus venosus valve, typically located freely in the right atrial cavity rather than focally attached to the fossa ovalis defect margin.^9^ Lambl excrescences are fine fibrous strands typically found on valve closure lines, not on the atrial septum.^10^ Atrial septal aneurysm presents as a diffuse saccular bulging of the entire fossa ovalis membrane with bidirectional excursion during the cardiac cycle, which has a distinct morphological appearance from the focal linear strand seen in this case.

Tricuspid valve lesions can also mimic fossa ovalis strands on limited imaging planes; as described by Roberts *et al*.,^14^ short linear lesions on tricuspid leaflets are located closer to the ventricle and are accompanied by abnormal transvalvular flow, requiring multiplanar TEE scanning to confirm anatomical origin.

### Incremental value of multimodal imaging

Transoesophageal echocardiography is the first-line imaging modality for evaluating atrial septal lesions, providing high-resolution anatomical detail of defect size, rim characteristics, and lesion morphology.^1^ However, TEE has inherent limitations in tissue characterization: it cannot reliably distinguish the internal composition of septal masses, assess microvascular perfusion, or differentiate benign fibrous tissue from pathological lesions, such as tumours or thrombi. In this case, initial TEE clearly identified the mobile septal structure but could not definitively exclude pathological masses, which was the clinical rationale for proceeding with further multimodal imaging.

Transthoracic contrast echocardiography adds critical information about tissue vascularity and perfusion.^11^ In this case, the absence of central contrast enhancement within the lesion directly demonstrated the lack of internal microvasculature, which effectively excluded highly vascular lesions, such as haemangioma and hypervascular tumours. The thin marginal annular enhancement reflected contrast pooling around the avascular fibrous structure rather than true tissue perfusion, which is a characteristic feature of benign septal remnants.

Contrast-enhanced cardiac CT provides complementary diagnostic value that extends far beyond simply confirming the ASD.^12^ First, CT allows comprehensive evaluation of the entire cardiac structure and mediastinum, excluding concurrent extracardiac lesions that may not be visualized by ultrasound. Second, CT density analysis and multi-phase enhancement patterns enable further differentiation of tissue components: the lesion in this case showed soft tissue density identical to normal septal myocardium, with no cystic change, calcification, or abnormal enhancement, further supporting a benign fibrous remnant rather than a neoplasm or thrombus. Third, CT provides accurate measurements of septal rim anatomy and thoracic vascular structure, which directly assists pre-procedural planning for transcatheter ASD closure.

Taken together, the multimodal approach progressively narrowed the differential diagnosis: TEE defined morphology and anatomical location; contrast echocardiography excluded vascular lesions; and CT confirmed tissue characteristics and excluded neoplastic and thrombotic pathologies. This stepwise workflow was essential to reach the final diagnosis of benign primary septal remnant and avoided unnecessary surgical resection of normal embryonic tissue.

### Clinical context and management implications

Residual fragments of the primary septum are relatively common anatomical variants in patients with fossa ovalis type secundum ASD, with a reported prevalence of up to 18% in adult ASD patients undergoing TEE evaluation.^14^ However, they are often under-recognized and frequently misdiagnosed as pathological masses, leading to unnecessary invasive procedures. Existing literature has described similar septal strands as incidental findings on TEE, but few reports have systematically discussed the multimodal differential diagnosis workflow combined with embryological principles.

This case carries important clinical implications for practice. First, when mobile septal strands are encountered in patients with ASD, primary septal remnant should be included as a primary benign differential diagnosis, and clinical reasoning should be grounded in embryological understanding rather than immediately assuming pathology. Second, a stepwise multimodal imaging strategy is recommended rather than relying solely on TEE, to improve diagnostic accuracy and avoid misdiagnosis. Third, confirmed primary septal remnants are benign lesions with no increased risk of thromboembolism or endocarditis and do not require separate resection during ASD repair or closure.^14^ In our case, the patient underwent transcatheter ASD closure without resection of the remnant, and 3-month follow-up confirmed an uneventful outcome with no adverse events.

For patients with this finding, long-term dedicated follow-up is generally not required after ASD correction. However, clinicians should explicitly counsel patients about the benign nature of the finding to alleviate anxiety related to the initial ‘intracardiac mass’ suspicion.

## Data Availability

All data supporting the findings of this case report are presented within the published article. No additional underlying datasets were generated or analysed during the current study.

## Lead author biography

The first author is pursuing a Master of Medicine degree at the Affiliated Asia Heart General Hospital of Wuhan University of Science and Technology, specializing in cardiac ultrasound.

## Notes

**Consent:** Written informed consent was obtained from the patient for publication of this case report and accompanying images, in full accordance with Committee on Publication Ethics (COPE) best practice guidelines. The patient was provided with a copy of the manuscript prior to providing consent.

## References

[1] Silvestry FE, Cohen MS, Armsby LB, Burkule NJ, Fleishman CE, Hijazi ZM, et al Guidelines for the echocardiographic assessment of atrial septal defect and patent foramen ovale: from the American Society of Echocardiography and Society for Cardiac Angiography and Interventions. J Am Soc Echocardiogr 2015;28:910–958. 10.1016/j.echo.2015.05.015 26239900

[2] Anderson RH, Brown NA, Webb S. Development and structure of the atrial septum. Heart 2002;88:104–110. 10.1136/heart.88.1.104 PMC1767197 12067964

[3] Hagen PT, Scholz DG, Edwards WD. Incidence and size of patent foramen ovale during the first 10 decades of life: an autopsy study of 965 normal hearts. Mayo Clin Proc 1984;59:17–20. 10.1016/s0025-6196(12)60336-x 6694427

[4] Geva T, Martins JD, Wald RM. Atrial septal defects. Lancet 2014;383:1921–1932. 10.1016/S0140-6736(13)62145-5 24725467

[5] Hrabak-Paar M, Hübner M, Štern-Padovan R, Lušić M. Hemangioma of the interatrial septum: CT and MRI features. Cardiovasc Intervent Radiol 2011;34 Suppl 2:90–93. 10.1007/s00270-010-0062-1 21153412

[6] Maleszewski JJ, Bois MC, Bois JP, Young PM, Stulak JM, Klarich KW. Neoplasia and the heart: pathological review of effects with clinical and radiological correlation. J Am Coll Cardiol 2018;72:202–227. 10.1016/j.jacc.2018.05.026 29976295

[7] Habib G, Lancellotti P, Antunes MJ, Bongiorni MG, Casalta JP, Del Zotti F, et al 2015 ESC guidelines for the management of infective endocarditis: the task force for the management of infective endocarditis of the European Society of Cardiology (ESC). Endorsed by: European Association for Cardio-Thoracic Surgery (EACTS), the European Association of Nuclear Medicine (EANM). Eur Heart J 2015;36:3075–3128. 10.1093/eurheartj/ehv319 26320109

[8] Murayama H, Tamaki S, Kato N, Yuji N, Yokote J, Mutsuga M, et al Right atrial myxoma associated with atrial septal defect: a case report and review of the literature. Ann Thorac Cardiovasc Surg 2001;7:166–169. 11481024

[9] Loukas M, Sullivan A, Tubbs RS, Weinhaus AJ, DerDerian T, Hanna M, et al Chiari’s network: review of the literature. Surg Radiol Anat 2010;32:895–901. 10.1007/s00276-010-0639-z 20198476

[10] Ammannaya GKK . Lambl’s excrescences: current diagnosis and management. Cardiol Res 2019;10:207. 10.14740/cr892 PMC6681848 31413776

[11] Senior R, Becher H, Monaghan M, Agati L, Zamorano J, Vanoverschelde JL, et al Clinical practice of contrast echocardiography: recommendation by the European Association of Cardiovascular Imaging (EACVI) 2017. Eur Heart J Cardiovasc Imaging 2017;18:1205–1205af. 10.1093/ehjci/jex182 28950366

[12] Sachdeva R, Armstrong AK, Arnaout R, Grosse-Wortmann L, Han BK, Mertens L, et al Novel techniques in imaging congenital heart disease: JACC scientific statement. J Am Coll Cardiol 2024;83:63–81. 10.1016/j.jacc.2023.10.025 PMC10947556 38171712

[13] Baumgartner H, De Backer J, Babu-Narayan SV, Budts W, Chessa M, Diller GP, et al 2020 ESC Guidelines for the management of adult congenital heart disease. Eur Heart J 2021;42:563–645. 10.1093/eurheartj/ehaa554 32860028

[14] Martin SS, Shapiro EP, Mukherjee M. Atrial septal defects–clinical manifestations, echo assessment, and intervention. Clin Med Insights Cardiol 2015;8:93–98. 10.4137/CMC.S15715 PMC4373719 25861226

